# The control of prickle formation in *Rubus*

**DOI:** 10.1093/plphys/kiag551

**Published:** 2026-07-28

**Authors:** Brian St. Aubin, Thomas J Poorten, Andrew Fister, Cherie Ochsenfeld, Joel Reiner, Allie Sandra Castillo, Rishi Aryal, Tomáš Brůna, Olga Dudchenko, Daniel James Sargent, Daniel Mead, Matteo Buti, Alexander Silva-Córdoba, Melanie Pham, David Weisz, Nahla Bassil, Hudson Ashrafi, Erez Lieberman Aiden, Nathaniel Graham, Raj Deepika Chauhan, Eric Dean, Warner Lowry, Lauren Redpath, Pradeep Marri, Shai J Lawit, Gina M Pham, Margaret Worthington, Brian C W Crawford

**Affiliations:** Research & Development, Pairwise, 807 E. Main Street Suite 4-100, Durham, NC 27701, United States; Research & Development, Pairwise, 807 E. Main Street Suite 4-100, Durham, NC 27701, United States; Research & Development, Pairwise, 807 E. Main Street Suite 4-100, Durham, NC 27701, United States; Research & Development, Pairwise, 807 E. Main Street Suite 4-100, Durham, NC 27701, United States; Research & Development, Pairwise, 807 E. Main Street Suite 4-100, Durham, NC 27701, United States; Research & Development, Pairwise, 807 E. Main Street Suite 4-100, Durham, NC 27701, United States; Department of Horticultural Science, Campus Box 7609, North Carolina State University, Raleigh, NC 27695-7609, United States; DOE Joint Genome Institute, Lawrence Berkeley National Laboratory, 1 Cyclotron Road, Berkeley, CA 94720, United States; The Center for Genome Architecture, Department of Biochemistry and Molecular Biology, University of Texas Medical Branch, 301 University Boulevard, Galveston, TX 77555, United States; Center for Theoretical Biological Physics, Rice University, BioScience Research Collaborative, 6500 Main Street, Suite 1005G, Houston, TX 77030, United States; Plant Genetics, National Institute of Agricultural Botany, 93 Lawrence Weaver Road, Cambridge CB3 0LE, United Kingdom; Wellcome Sanger Institute, Wellcome Genome Campus, Hinxton, Cambridge CB10 1SA, United Kingdom; Owlstone Medical Ltd, 183 Cambridge Science Park, Milton Road, Cambridge CB4 0GJ, United Kingdom; Department of Agriculture, Food, Environment and Forestry (DAGRI), University of Florence, Piazzale delle Cascine 18, 50144 Florence, Italy; Department of Horticulture, University of Arkansas, 316 Plant Sciences Building, Fayetteville, AR 72701, United States; The Center for Genome Architecture, Department of Biochemistry and Molecular Biology, University of Texas Medical Branch, 301 University Boulevard, Galveston, TX 77555, United States; Center for Theoretical Biological Physics, Rice University, BioScience Research Collaborative, 6500 Main Street, Suite 1005G, Houston, TX 77030, United States; The Center for Genome Architecture, Department of Molecular and Human Genetics, Baylor College of Medicine, One Baylor Plaza, Houston, TX 77030, United States; USDA-ARS, National Clonal Germplasm Repository, 33447 Peoria Road, Corvallis, OR 97333, United States; Department of Horticultural Science, Campus Box 7609, North Carolina State University, Raleigh, NC 27695-7609, United States; The Center for Genome Architecture, Department of Biochemistry and Molecular Biology, University of Texas Medical Branch, 301 University Boulevard, Galveston, TX 77555, United States; Center for Theoretical Biological Physics, Rice University, BioScience Research Collaborative, 6500 Main Street, Suite 1005G, Houston, TX 77030, United States; Research & Development, Pairwise, 807 E. Main Street Suite 4-100, Durham, NC 27701, United States; Research & Development, Pairwise, 807 E. Main Street Suite 4-100, Durham, NC 27701, United States; Research & Development, Pairwise, 807 E. Main Street Suite 4-100, Durham, NC 27701, United States; Research & Development, Pairwise, 807 E. Main Street Suite 4-100, Durham, NC 27701, United States; Research & Development, Pairwise, 807 E. Main Street Suite 4-100, Durham, NC 27701, United States; Research & Development, Pairwise, 807 E. Main Street Suite 4-100, Durham, NC 27701, United States; Research & Development, Pairwise, 807 E. Main Street Suite 4-100, Durham, NC 27701, United States; Research & Development, Pairwise, 807 E. Main Street Suite 4-100, Durham, NC 27701, United States; Department of Horticulture, University of Arkansas, 316 Plant Sciences Building, Fayetteville, AR 72701, United States; Research & Development, Pairwise, 807 E. Main Street Suite 4-100, Durham, NC 27701, United States

## Abstract

Prickles on blackberry and raspberry canes make pruning, harvesting, and handling more difficult and can increase labor costs for growers. The trait has been challenging to improve in these clonal crops because it is recessive and linked to undesirable agronomic traits. In blackberry and red raspberry, breeding programs have used recessive mutants at the *S* locus to generate prickleless cultivars for the last century. In this study, we identified independent loss-of-function mutations in a WUSCHEL-LIKE HOMEOBOX transcription factor, *WOX1*, as the genetic basis of the prickleless *S* locus in both blackberry and red raspberry. We mapped the *S* locus using integrated genome-wide association, bulked segregant analysis, and identity-by-descent analyses informed by breeding pedigrees. Additionally, we generated a genome sequence from Luther Burbank's prickleless blackberry variety Burbank Thornless that contained an additional allele of *WOX1*. To verify the gene's role, we used gene editing to knock out *WOX1* in an elite prickled commercial blackberry line. All edited plants were prickleless and lacked glandular trichomes, confirming that *WOX1* controls a joint developmental pathway. Other plant traits were unchanged, indicating *WOX1* is a specific and safe target for improvement. Gene editing can enable breeders to remove prickles directly from elite varieties, reducing the need for extensive breeding cycles and delivering safer, easier-to-harvest cultivars to growers.

## Introduction

In *Rubus* species like blackberry and red raspberry, prickles serve as a key defensive feature that protects the plant from herbivores and other threats. These sharp outgrowths, which arise from the nonvascular tissue, act as a physical barrier, deterring animals from feeding on or damaging the plant's stems and leaves (Table 1). In addition to providing protection from grazing animals, prickles can also reduce damage from mechanical stress, such as wind or contact with other plants. While prickles are beneficial for the plant's survival in the wild, they pose challenges in agricultural settings, particularly during harvesting and pruning, when they can hinder manual handling and increase labor efforts (Darrow 1928; Jennings 1986, 1988). Because of this, breeding efforts in *Rubus* crops, collectively known as caneberries, often focus on developing prickle-free cultivars to improve efficiency and ease of cultivation (Finn and Clark 2011).

**Table 1:** Differences in epidermal outgrowths.

Structure	Botanical ontology	Vasculature	Model
Trichomes	Epidermally derived	No vasculature	Arabidopsis/cotton/others
Glandular trichomes	Epidermally derived	No vasculature	Cucumber/tomato/other
Prickles	Epidermally or cortex derived	No vasculature	*Rosa*/*Rubus*
Spines	Modified leaves	Vasculature	Cacti
Thorns	Modified branches	Vasculature	Citrus

In *Rubus* species, both glandular trichomes and prickles are epidermal outgrowths that serve defensive roles, though they differ in structure and function (Khadgi and Weber 2020a). Glandular trichomes are typically smaller, hairlike structures with secretory cells at their tips (distal heads) that produce compounds such as oils, terpenoids, or phenolics, which deter herbivores and protect against pathogens (Werker 2000). In contrast, prickles are larger, sharp outgrowths that physically defend the plant by preventing herbivores or other animals from feeding on the stems or leaves. While glandular trichomes primarily offer chemical defense, prickles provide mechanical protection. Interestingly, both structures develop from the epidermis, and the genetic mechanisms underlying their formation may overlap. Studies in *Rubus* suggest that similar regulatory pathways could influence the development of both glandular trichomes and prickles, highlighting a potential genetic connection between chemical and physical defenses in the plant (Coyner et al. 2005). Understanding this relationship can aid in breeding efforts, especially for prickle-free cultivars, without compromising the plant's natural defenses provided by glandular trichomes.

The genus *Rubus*, which is part of the Rosaceae family, is an ideal model for investigating the mechanisms behind prickle formation and development. The concept of prickleless canes in red raspberries (*Rubus idaeus*) was first documented in 1629 by John Parkinson in *Paradisi in sole paradisus terrestris* (Parkinson 1629; Jennings 1988). Breeding for prickleless (also referred to as thornless) plants in caneberries has a long history that is driven by the need to improve harvesting efficiency and reduce labor costs associated with handling prickled plants. The quest for prickleless caneberry cultivars began in the early 20th century, with efforts focused on identifying naturally occurring prickleless mutations and incorporating them into breeding programs. In raspberries, the development of prickleless cultivars gained momentum with the discovery of the allele “spineless' ('*s^ri^*”, *R. idaeus*) in prickleless types of the progeny of the Scottish variety Burnetholm (Lewis 1939). Subsequent breeding using this “*s*” allele led to the creation of all the modern prickleless red raspberry varieties like Glen Ample and Joan J. (Jennings 1986).

Prickleless blackberry (*Rubus* subgenus *Rubus*) cultivars have been developed from multiple heritable and nonheritable mutations (Clark et al. 2007). Many early prickleless cultivars, including Thornless Evergreen, Thornless Loganberry, and Thornless Youngberry, were found to be chimeral, with the prickleless alleles only present in the nonheritable epidermal (L1) layer, complicating their use in breeding. A dominant, heritable source of prickleless canes was discovered in the octoploid cultivar Austin Thornless, but its use in modern breeding programs has been limited by its partial sterility, trailing habit, and presence of prickles in the basal 30 cm of canes. The recessive “*s*” allele (the “*s^ru^*”, *Rubus ulmifolius* allele) is by far the most used source of the prickleless trait in blackberry. The *s^ru^* allele is derived from the diploid *R. ulmifolius* var. *inermis*, also known as *Rubus rusticanus* var. *inermis*. Breeders generated a prickled tetraploid *R. ulmifolius* var. *inermis* cultivar, John Innes, carrying the recessive “*s*” allele, from an unreduced germ cell (Crane and Darlington 1927). The prickleless blackberry cultivar Merton Thornless was developed in the 1930s from a backcross of a prickleless F_2_ seedling to blackberry cultivar John Innes, and the *s^ru^* allele then served as a foundation for modern prickleless blackberry breeding (Crane and Darlington 1927; Coyner et al. 2005). Luther Burbank's most famous prickleless blackberry was a diploid variety of *R. ulmifolius* known as Burbank Thornless, which he promoted as an easier-to-handle alternative to traditional prickled varieties (Butterfield 1928). Subsequent breeding advancements using Merton Thornless led to popular thornless blackberry varieties such as Navaho, Apache, Triple Crown, and Chester, all of which offer ease of cultivation and improved fruit quality (Coyner et al. 2005). These prickleless cultivars have become valuable in commercial production, significantly reducing the challenges associated with harvesting and field management while maintaining high fruit quality.

The *s^ru^* gene responsible for prickleless canes in blackberry was determined to be allelic with the *s^ri^* gene in raspberry (Jennings 1986). Both *s^ru^* and *s^ri^* have been mapped to overlapping regions on *Rubus* chromosome 4 (Castro et al. 2013; Khadgi and Weber 2020b; Johns et al. 2025). There are a few limitations to using the *s^ru^* gene for achieving prickleless canes in blackberry breeding including recessive inheritance and associations with undesirable plant and berry traits. Modern fresh-market blackberry cultivars are tetraploids with multisomic inheritance. Therefore, 4 copies of the recessive *s^ru^* allele are required for a plant to develop the prickleless phenotype. Early breeders using the *s^ru^* allele conducted self-pollination, sib-crossing, and backcrossing to develop prickleless plants, all of which contributed to inbreeding and loss of diversity around the *s^ru^* locus. This loss of diversity is still evident in modern breeding programs; an extensive linkage disequilibrium block was identified around the *s^ru^* locus in an association panel of fresh-market blackberry cultivars and breeding selections composed of 38 prickled and 336 prickleless genotypes (Johns et al. 2025). Merton Thornless has several shortcomings as a cultivar, including susceptibility to freeze damage, trailing growth habit, late harvest season, large seed size, and high fruit acidity. Many of these traits appear to be in linkage with the *s^ru^* locus, and breaking up deleterious linkages has been challenging, particularly considering the recessive inheritance of the *s^ru^* allele and extensive linkage disequilibrium around the prickleless locus (Clark 2005).

Gene editing offers a solution to the problems associated with using the *s^ru^* allele in breeding programs by allowing precise genetic modifications, eliminating unwanted traits without affecting favorable ones (Moore 1984). Using this technology, breeders could modify the prickled allele(s) at the *s^ru^* locus in an elite prickled variety with exceptional fruit quality and agronomic traits without the time and expense of further crossing and the complications of potential inbreeding and linkage drag around the locus. This technology accelerates the breeding process and improves overall crop performance (Fister et al. 2024).

In this paper, we identify mutations in a WUSCHEL-LIKE HOMEOBOX-type (WOX) transcription factor responsible for the prickleless phenotype in red raspberry and blackberry. We developed a new genome assembly and annotation for *R. ulmifolius* Burbank Thornless and mapped the “s” locus using a combination of genome-wide association studies (GWAS), bulked segregant analysis (BSA), and family-based identity-by-descent (IBD) mapping. We also developed a transformation protocol for blackberry and confirmed the target gene through gene editing, successfully introducing the prickleless phenotype into a commercial blackberry variety previously featuring prickles.

## Results

### Generating a diversity panel to understand the prickle trait in *Rubus*

**Figure 1:**
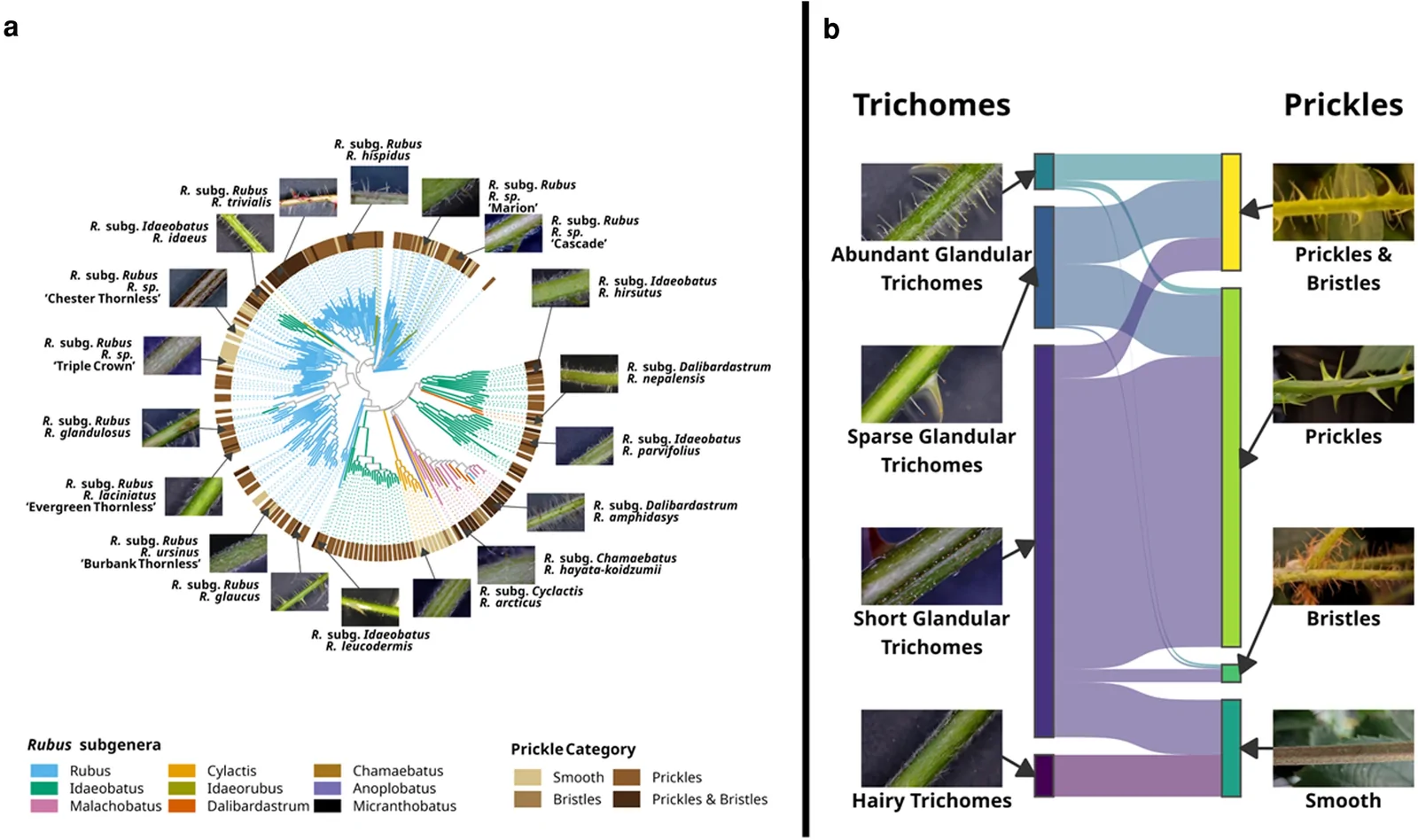
Morphological and genetic variation within the *Rubus* population, illustrating the comparative morphology of trichomes, glandular trichomes, and prickles. Panel a includes representative photographs showing the diversity of epidermal outgrowths on *Rubus* canes, as observed by eye. Panel b includes representative images of the diversity of trichome, glandular trichome, and prickle presentations as viewed under a microscope. Colored arrows indicate different epidermal structures: yellow = simple trichomes, red = short glandular trichomes, green = elongated glandular trichomes, blue = prickles. The scoring system indicates a wide range of variability within the panel, with a subset of individuals exhibiting both prickle-free and glandular trichome traits. The panels are intended to illustrate qualitative prickle diversity, and scale bars are therefore not included.

We surveyed a diverse panel of 268 *Rubus* accessions, including 224 prickled and 44 prickleless accessions from 8 subgenera, ensuring a broad representation of genetic and phenotypic variation for the prickle trait (Fig. 1a; Table S1). We observed a prickleless (smooth) phenotype in some *Rubus* subg. *Rubus* and all *Rubus* subg. *Cylactis*. Prickleless red raspberries (*Rubus* subg. *Idaeobatus*) have been identified and bred, but none were included in the panel. Accessions were phenotyped with both unaided observation of the cane and with the use of a dissecting microscope (Fig. 1a). Using qualitative visual evaluation, we distinguished 4 classes of phenotypes: plants with sharp, lignified prickles, plants with elongated, hairlike bristles, plants with both hairlike bristles and prickles, and smooth plants that lacked any elongated epidermal structure (Table 2). When viewed with a dissecting microscope, we were able to better classify trichome types. Most plants had glandular trichomes, which varied in size and density. Some accessions had 2 unique glandular trichome structures: one short (<1 mm) outgrowth, and one with an elongated stalk. With the microscope we were able to discern that the bristles observed by eye were these elongated glandular trichomes. All accessions had simple trichomes, which also varied in density among accessions. These typically laid flat along the cane, which sometimes made them difficult to observe without a microscope. In our study, we observed a Cramer's V association statistic of 0.47 indicating a moderate correlation between the coded values for glandular trichomes and the type of prickles across the *Rubus* diversity panel. Varieties that exhibited prickles had glandular trichomes, suggesting a possible linkage or shared developmental pathway between these 2 epidermal structures (Fig. 1b). Additionally, a subset of subgenus *Rubus* varieties that were prickle-free lacked glandular trichomes entirely, which reinforced the idea that the absence of prickles may be genetically or developmentally associated with the absence of glandular trichomes. This pattern suggests a potential genetic basis underlying the co-occurrence of these traits, which could be crucial for understanding the mechanisms that regulate epidermal outgrowths in *Rubus* species.

**Table 2:** Prickle/trichome relationship in diversity panel.

	Prickle rating				Total
	Smooth	Bristles	Prickles	Prickles & bristles	
Trichome rating					
Hairy trichomes	42	6	0	0	48
Short glandular trichomes	17	17	132	17	183
Short and elongated glandular trichomes (not abundant)	0	1	63	21	85
Short and elongated glandular trichomes (abundant)	0	1	3	12	16
Total	59	25	198	50	332

The inheritance of the “spineless” gene in red raspberry and blackberry has been well documented through traditional breeding studies. In red raspberry, the *s^ri^* allele was successfully incorporated into various commercial cultivars. Within the diversity panel we identified both prickled and prickleless related red raspberry lines that were descended from crosses using the *s^ri^* allele (Figure S1a). Similarly for blackberry, we identified samples within our diversity panel that contained the *s^ru^* allele from the original Merton Thornless accession (Figure S1b). To aid the identification of the *S* locus we analyzed samples within our diversity panel that contained either of the *s^ru^* and *s^ri^* alleles (Figure S1;  Tables S2 and S3) through a combination of GWAS, BSA, and IBD analyses.

### Developing a Burbank Thornless genome assembly and annotation

Like the progenitor of Merton Thornless, Burbank Thornless is also a prickleless diploid *R. ulmifolius,* but the providence of this line was unknown. Luther Burbank may or may not have obtained the same prickleless genotype used to introgress the *s^ru^* allele into modern blackberry breeding programs. To gain genomic insight into the basis of the prickleless trait, we generated a chromosome-scale assembly of Burbank Thornless using Pacific Biosciences long-reads scaffolded with high-throughput chromosome conformation capture (Hi-C). The final assembly comprised 342 Mb across 288 scaffolds, including 7 chromosome-length scaffolds totaling 299 Mb (87% of the genome). Structural annotation predicted 29,708 protein-coding genes and 38,105 coding transcripts, with an average gene length of 3.4 kb (Table 3). Benchmarking with BUSCO identified 96.2% of conserved eudicot genes, and most loci were further supported by Expressed Sequence Tag alignments, peptide homology, and high annotation confidence scores (C-scores, which reflect the strength of external evidence supporting each gene model). Functional annotation assigned putative functions to most transcripts across multiple databases and domain resources. Comparative analyses revealed strong collinearity with the *Rubus argutus* “Hillquist” genome and with other published diploid *Rubus* genomes, reflecting conserved chromosome structure across the genus (Brůna et al. 2023). Notably, the Burbank Thornless gene set is smaller but more completely annotated than those of other *Rubus* genomes, reducing the likelihood of fragmented or spurious predictions and thereby enhancing its utility for candidate gene discovery. Complete methods and results describing the genome assembly and annotation are provided in File S1 and Supplemental Tables S4 and S5.

**Table 3:** Summary statistics for the assembled *R. ulmifolius* cv. Burbank Thornless genome.

Estimated genome size (flow cytometry)	366.75 Mb
Total assembly length	341.59 Mb
No. scaffolds	288
No. chromosomes	7
Size of sequence anchored on chromosomes	299 Mb
Maximum scaffold length	52.4 Mb
N50 scaffold length (bp)	41.1 Mb
Repeat content	48.47%
No. protein-coding genes	29,708
No. protein-coding transcripts	38,105
Average coding sequence length	3,405 bp
BUSCO genes (eudicots_odb10)	96.2%

### Fine mapping the prickleless causal mutation at the *S* locus

Three different mapping strategies were applied to resolve the causal locus. First, GWAS was used to find the locus associated with prickles on the whole diversity panel and on subsets of the diversity panel based on prior knowledge of the potential source of the causal allele. We also generated a segregating population for BSA in blackberry. Finally, we used pedigrees to inform an IBD analysis to further refine the causal locus and manually inspected sequence variation in red raspberry and blackberry lines to look for recessive mutations in annotated genes.

**Figure 2:**
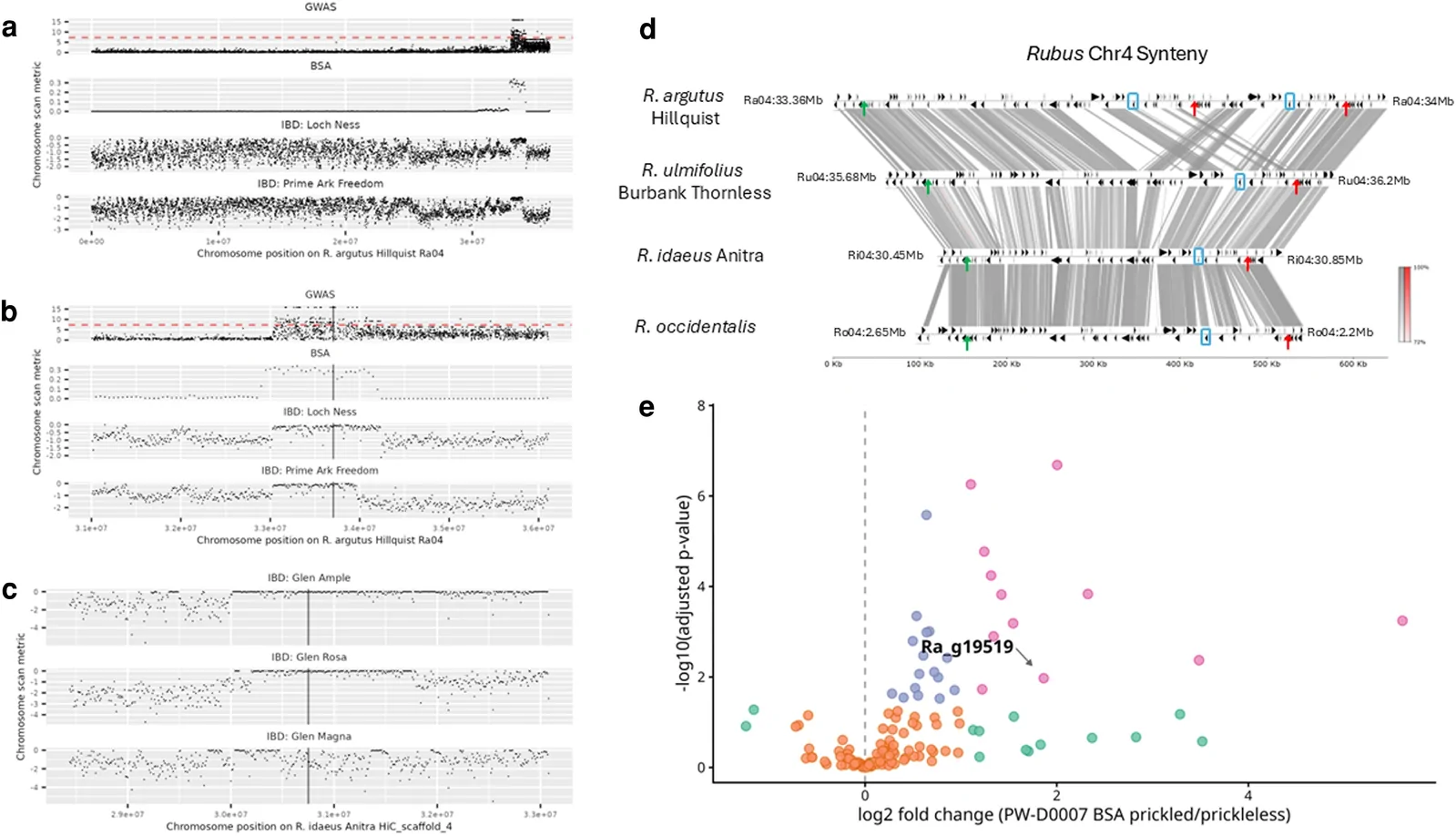
Fine mapping of *s* allele on *Rubus* chromosome 4. a) *Rubus* chromosome 4 SNP density plots for the GWAS, bulk segregant analysis (BSA), and example individual lines mapped to the *R. argutus* cv. Hillquist genome. b) Zoomed-in view of 5-Mb region centered on pricklelessness-associated region on chromosome 4 in *R. argutus* Hillquist genome. c) Zoomed-in view of 5-Mb region centered on syntenic pricklelessness-associated region on chromosome 4 in the *R. idaeus* cv. Anitra (red raspberry) genome. GWAS panels show −log10(*p*) on *y* axis; dashed red line shows standard significance threshold *P* = 5 × 10^−8^. BSA panels show number of filtered variants per 100-kbp window with a 50-kb step size on the *y* axis. Identity-by-descent (IBD) panels show a metric to identify regions of excess homozygosity on *y* axis (–[# heterozygous sites per 100-kb window/median of # heterozygous sites per 100-kb window]). d) Synteny plot showing homologous pricklelessness-associated region in 4 *Rubus* genomes. Grey bands show mummer alignments (≥100 bp and ≥70% identity). Black arrows correspond to gene locations. Green arrows show syntenic positions of left border, and red arrows show syntenic positions of right border. Blue box shows locations of syntenic *WOX1* gene models. The region between left and right boundary in *R. argutus* is Ra04:33,393,466 to 33,774,434 (Ra04:33,951,000 is right border when including tandem duplication misassembly); in *R. ulmifolius* is Ru04:35,726,940 to 36,153,835; in *R. idaeus* is Ri04:30,482,969 to 30,807,700; in *R. occidentalis* is Ro04:2,224,711 to 2,591,926. For visual clarity of synteny, the *R. occidentalis* chromosome 4 was reverse-complemented prior to alignment and plot generation. The grey and red scale bars show alignment sequence similarity for forward and reverse alignments, respectively. e) Gene expression volcano plot for genes in the IBD interval, PW-D0007 siblings segregating for prickles. Red circle highlights *WOX1*.

We used a SNP-based GWAS to investigate the genetic basis of prickle development in a *Rubus* population of 43 accessions using the software package GWASpoly (Voichek and Weigel 2020; Lemane et al. 2022). We scanned for associations between the presence of a homozygous mutant allele and the presence of the prickleless phenotype and were able to identify regions of the genome potentially linked to the *S* gene. We found that the leave-one-chromosome-out (LOCO)method to control for population structure had negligible effect on association modeling by visual assessment of the QQ-plots (Figure S2), and proceeded to investigate GWAS results with the base association model. We identified a region significantly associated with the prickleless trait at the end of chromosome Ra04 between 33,000,000 bp and the end of chromosome at 36,116,165 on the *R. argutus* genome (35,312,865 to 38,546,343 bp in *R. ulmifolius*) (Fig. 2a, b; Figure S2, S3). This region overlaps with another region significantly associated with the prickleless trait that was recently identified in a GWAS panel of 374 fresh-market blackberry genotypes from 30.48 to 36.04 Mb on chromosome Ra04 (Johns et al. 2025).

To further refine the candidate region, we generated a segregating population by self-crossing PW-D0007, a line triplex for the *s* allele (*Ssss*). The resulting progeny displayed either the prickleless (32 individuals) or prickled phenotype (65 individuals), which fits the expected segregation ratios for a double simplex cross under both random chromosome (χ^2^ = 3.30; *P* = 0.07) and random chromatid assortment (χ^2^ = 0.87; *P* = 0.35). We used this population in a BSA. High-throughput sequencing was then performed on both groups, generating genome-wide sequence data representing the genetic makeup of the prickleless and prickled groups (Fig. 2a, b).

Given the tetraploid nature of our blackberry genotypes, we accounted for the complexity of allele dosage, where individuals can have varying numbers of copies of a given allele (eg 0 to 4 copies). To handle this complexity, we focused on identifying regions of the genome where allele frequencies significantly differed between the 2 bulks. By comparing the allele frequencies in the prickled versus prickleless bulks, we were able to pinpoint the genomic region enriched for prickleless alleles to a 1.3-Mb region on chromosome Ra04 between 32,950,000 and 34,250,000 bp (35,259,235 to 36,474,787 in *R. ulmifolius*) (Fig. 2 a, b).

In our efforts to fine-map the locus responsible for the *S* gene, we also utilized a family-based IBD analysis. We selected a subset of prickleless and prickled individuals from blackberry and raspberry along with prickled black raspberry, focusing on individuals known to have the *s^ru^* or s*^ri^* alleles to maximize shared genomic regions inherited from common ancestors (Figure S1a,b). Using genotype information from the GWAS resequencing data, we identified regions of extended homozygosity in individuals without prickles and in individuals with prickles. By comparing these shared homozygous regions across the prickleless population, we were able to identify genomic intervals potentially harboring the gene of interest. We used both red raspberry and blackberry alleles to narrow the region further. We compared SNPs in heterozygous and homozygous individuals in a 20-kb region near the borders of the locus (Fig. 2 a, b; Table 4). We identified that the prickleless cultivar Prime-Ark Freedom had a recombination event that narrowed the region of homozygosity on the left side to 33,286,753 bp on the *R. argutus* cv. Hillquist chromosome Ra04 (35,349,585 to 36,170,216 in *R. ulmifolius*) (Table 4). The *R. argutus* cv. Hillquist genome contains a 166-kb duplication within the region, impacting the identification of the right border. To resolve this issue both the *Rubus occidentalis* v3 and *R. idaeus* cv. Anitra genomes were primarily leveraged to identify SNPs (VanBuren et al. 2016; Davik et al. 2022). The right border was set at Ri04:30,807,700 which corresponds to Ra04:33,951,000 and 33,774,434 in the *R. argutus* cv. Hillquist genome owing to the duplication (Fig. 2d). This narrowed region contained 82 to 140 predicted annotated genes depending on the genome and annotation. In red raspberry, the prickleless Glen Ample line shared homozygosity within the previous identified region with the related prickled line Glen Magna. This identified SNPs that provided a discrete boundary within the region at position 30,482,969 on chromosome 4 in the *R. idaeus* cv. Anitra genome (corresponding with chromosome Ra04, position 33,393,466 in *R. argutus* cv. Hillquist and 35,726,846 in *R. ulmifolius*) (Fig. 2c). This reduced the region down to around 324 kb containing 62 to 115 gene models (Table S6).

**Table 4:** Genetic intervals potentially containing the causal mutation for the prickleless phenotype identified through fine mapping.

Source allele	Method	Change	Left/right border relative to Hillquist genome	*R. argutus* cv. “Hillquist” location (bp)	*R. ulmifolius* Burbank Thornless location (bp)	*R. occidentalis* ORUS 4115-3 location (bp)	*R. idaeus* cv. Anitra1611 location (bp)	Nearest gene (inside)
Red raspberry	Family-based analysis	A>T homozygous to heterozygous	Left	Ra04:33,393,466	Ru04: 35,726,846	Ro04:2,591,816	Ri04:30,482,969	Ra_g19461, Ruulm.4G300300, Ro04_G00141, Ri04_g21576
Blackberry	Family-based analysis	A>G	Left	Ra04:33,286,753	Ru04:35,604,782	Ro04:2,714,466	Ri04:30,373,221	Ra_g19440, Ruulm.4G298200, Ro04_G00118, Ri04_g21556
Blackberry	Family-based analysis	T>C	Right	Ra04:33,951,000 and 33,775,434	Ru04:36,151,812	Ro04:2,223,785	Ri04:30,807,700	Ra_g19578 and Ra_g19536, Ruulm.4G307000, Ro04_G00205, Ri04_g21637

### Expression analysis of candidate gene targets

To gain a better understanding of biological pathway differences in prickled and prickleless canes, we used transcriptome analyses to investigate gene expression differences in these 2 groups. This information aided in the evaluation of candidate genes from the locus. We generated transcriptome data from the PW-D0007 blackberry self-crossed population that was used for BSA, which included samples from plants segregating for the prickleless and prickled phenotypes. The dataset comprised meristem samples from growing canes with the intention of having part of the sample contain cells that are transitioning to prickles. We used nonbulked samples from 3 prickleless and 9 prickled plants. This approach aimed to identify differentially expressed genes linked to the presence or absence of prickles, with several genes also found within the IBD region. The results provided extra support for SNPs within genes associated with all *s* alleles.

Expression analysis revealed 2,357 upregulated genes in the prickled samples and 1,921 downregulated in the prickled samples compared to the prickleless samples (*P*adj <0.05) (Table S7). Within the region there were several genes that had higher expression in the prickled samples including Peptidase S10 Ra_g19503, Homeobox Ra_g19519, C2 domain Ra_g19534, Phosphoribulokinase/uridine kinase Ra_g19555, SLC26A/SulP transporter Ra_g19557, and C2 domain Ra_g19577 (Table 5; Fig. 2e). No genes within the region had lower expression in the prickled samples compared to the prickleless samples.

**Table 5:** Differential gene expression within interval.

Gene_ID	Start	Stop	Strand	Gene info	Domain info	PW-D0007 prickled/prickleless fold change
**Ra_g19503**	33,629,274	33,630,632	–		Peptidase S10	2.00
**Ra_g19519**	33,705,382	33,707,440	–	Indels in thornless genomes	Homeobox domain	1.86
**Ra_g19534**	33,761,529	33,762,047	–		C2 domain	1.24
**Ra_g19555**	33,838,869	33,850,045	+		Phosphoribulokinase/uridine kinase	1.54
**Ra_g19557**	33,853,606	33,854,114	–	Gene model error in first, second and third exons	SLC26A/SulP transporter	3.48
**Ra_g19577**	33,943,104	33,943,622	–		C2 domain	1.22

### Identification of the *S* gene

To understand the potential causal genes within the region, we attempted to use computational genome annotation, which has become instrumental in exploring gene functions across the numerous new genomes. However, standard genome annotation poses challenges in making refinements based on new data or integrating valuable user input. Additionally, despite apparent synteny, sequence variations between *Rubus* species complicated alignment to a single reference genome, requiring a multigenome approach.

To address these challenges, we inspected gene models within the target interval and compared them to gene expression data for red raspberry, black raspberry, and blackberry. This comparison was facilitated by aligning 3 separate genome browsers, simultaneously allowing us to inspect SNPs from various whole genome sequencing efforts in the context of several genomes. Using the *R. argutus* cv. Hillquist, *R. occidentalis* v3 genome, and the *R. idaeus* cv. Anitra genome from rosaceae.org as references, we confirmed comparable genomic regions. Using the pyGenomeViz package with BLAST alignments and default parameters (https://github.com/moshi4/pyGenomeViz), we aligned the following regions: Ru04:35300000:36300000 (+), Ri HiCscaffold_4:30206071-30307189 (+), Ro04:1900000-3050000 (−), and Ra04:33000000-34000000 (+) (Fig. 2d). While some inconsistencies were noted in gene models across these genomes, the coding sequences for most expressed genes appeared correctly annotated. After finalizing the interval via IBD analysis, we made manual adjustments to the gene models within this region to increase annotation accuracy.

**Figure 3:**
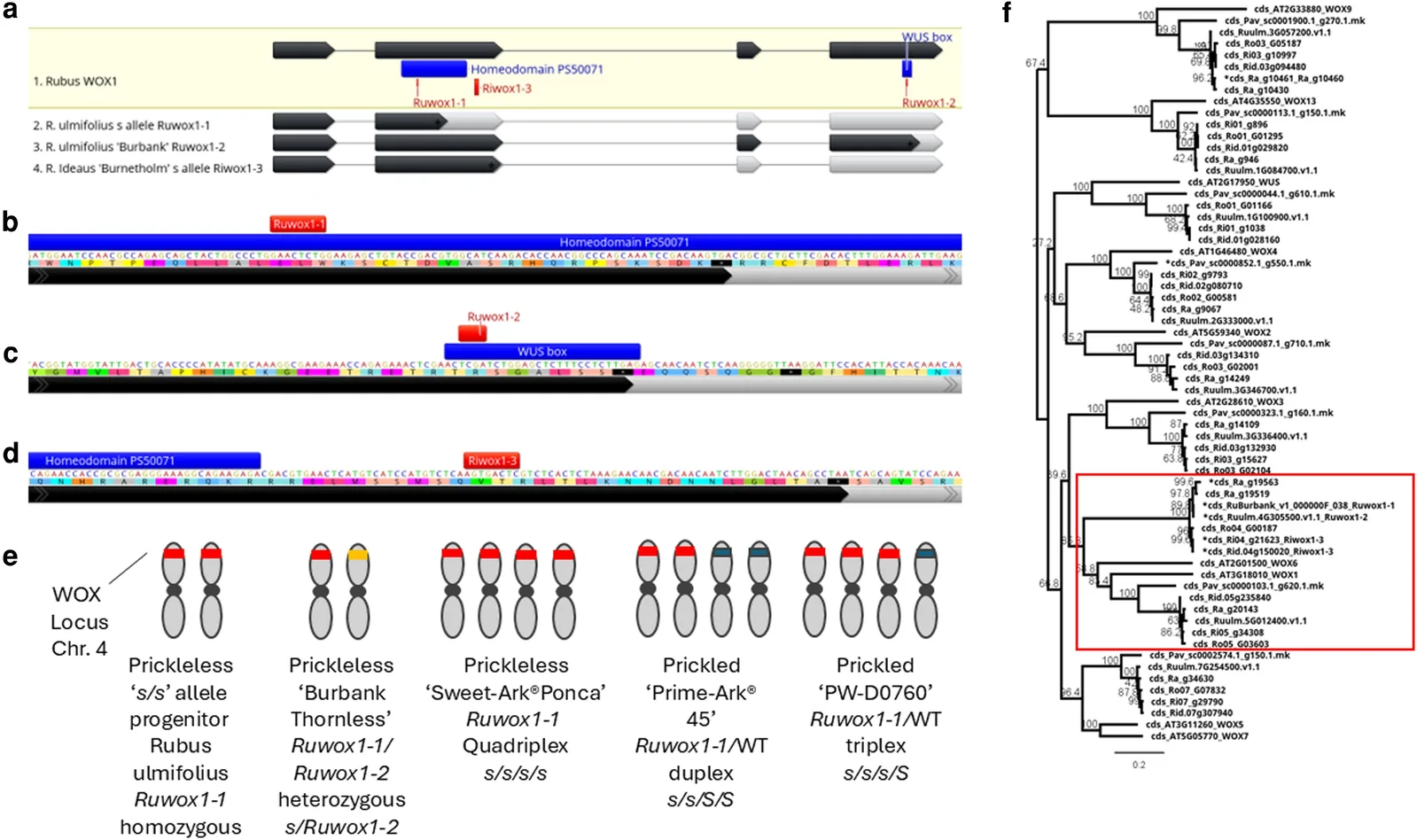
Mutant alleles and *WOX* gene phylogeny. a) *Rubus* wild-type *WOX1* gene with dark grey showing coding sequence with regions labeled that encode for homeobox and WUS box protein domains. The *s* alleles are labeled with light grey showing protein truncation due to premature stop codons. b–d) Close-up of alleles with red indicating the insertions leading to premature stop codons. e) Zygosity of blackberry *s* alleles in blackberry lines. Red indicates *s* allele; orange indicates allele from Burnbank Thornless, and blue indicates wild-type *S* allele. f) Phylogenomic tree of the conserved coding sequences (including indels) form closely related sequences to *WOX1*. *WOX13* and *WOX9* are included as an outgroup. The *WUS* sequence was not found in the *R. argutus* genome assembly and is substituted by the sequence from *R. ulmifolius*. The *WOX2* sequence was not found in the *R. idaeus* cv. Anitra genome assembly. Sequences with stop codon inducing indels are marked with an *. g) Arabidopsis *WOX* naming and the associated functions based on mutants and gene expression.

Through this refined approach, we identified an 8-bp insertion in the second exon of the Ra_g19519 (Ruulm.4G305500) gene within the prickleless haplotype in blackberry (Fig. 3a, b). Ra_g19519 encodes a WUSCHEL-Related Homeobox (WOX) protein, which is vital for balancing stem cell maintenance and differentiation within meristems in plants. In Arabidopsis, members of the *WOX* gene family have overlapping roles in various meristem regions, where they contribute to the development of plant lateral organs (Haecker et al. 2004; Park et al. 2005; Wu et al. 2005; Breuninger et al. 2008; Shimizu et al. 2009; Nakata et al. 2012; Qian et al. 2021) (Table 6). Phylogenetic analysis aligned Ra_g19519 (and its black raspberry ortholog Ro04_G00187) with the Arabidopsis *WOX1* gene (At3g18010). For simplicity, we designated Ra_g19519 as *RuWOX1* (Fig. 3f).

**Table 6:** Reported function of *WOX* gene family members.

*WOX* family members	Reported function	Reference
*WUS*	Shoot apical meristem	van der Graaff et al. (2009); Ikeda et al. (2009)
*WOX1*/*WOX3*	Lateral organ formation; prickles in eggplant	Zhang et al. (2011); Qian et al. (2021)
*WOX2*/*WOX5*	Root apical meristem	Breuninger et al. (2008)
*WOX6*	Gametophyte	Park et al. (2005)
*WOX5*/*WOX7*	Adventitious roots	van der Graaff et al. (2009)
*WOX2*/*WOX8*/*WOX9*	Embryonic patterning/carpel	Haecker et al. (2004); Wu et al. (2005)

The 8-bp insertion, termed *Ruwox1-1*, co-segregated with the prickleless (*s^ru^*) allele across all examined populations (Fig. 3e). The insertion introduces a premature stop codon within the annotated homeodomain of *RuWOX1*, likely disrupting its function (Fig. 3b). During IBD analysis of the blackberry *s^ru^* allele, we encountered difficulties in reconciling the prickle-free phenotype of the Burbank Thornless cultivar, which appeared heterozygous for the *Ruwox1-1* allele. Closer examination of the second Ra_g19519 allele in Burbank Thornless (unassembled contig 000000F_038) revealed 2 distinct haplotypes at the *S* locus, leading to the discovery of an additional allele, *Ruwox1-2*. *Ruwox1-2* carries a 4-bp insertion in the WUS box, a conserved motif critical to the function of *WOX* family genes (Fig. 3c). This finding indicates that Burbank Thornless has a compound heterozygous genotype at the *S* locus, with one copy of *Ruwox1-1* and one of *Ruwox1-2* (Fig. 3e). Because the Burbank Thornless assembly is not phased, only one allele at the *S* locus was retained in the final reference sequence. By chance, the assembled haplotype corresponds to *Ruwox1-2*, the allele that was not introgressed into domesticated germplasm, while the alternative allele (*Ruwox1-1*) segregates with the prickleless trait in modern breeding populations. In contrast, our prickled commercial line, PW-D0760 (Adams 2022), was found to carry the *Ruwox1-1* allele in triplex, retaining one functional copy alongside 3 *s^ru^* alleles. Further analysis of Ri04_g21623, the red raspberry orthologue of Ra_g19519, identified an 8-bp insertion just downstream of the homeodomain that introduces a premature stop codon before the last 2 exons (Fig. 3d). We named this allele *Riwox1-3*, and it segregated with the prickleless (*s^ri^*) allele in red raspberry populations derived from the original Burnetholm cultivar. While evaluating phenotypic diversity across *Rubus* accessions, we observed that most *Cylactis* species exhibited a smooth phenotype. This observation prompted an investigation into potential *WOX* allele variation within the clade. Analysis of sequences orthologous to *Ra_g19519* from members of the subgenus *Cylactis* (*Rubus arcticus*, *R. lasiococcus*, and *Rubus pubescens*) identified nonsynonymous amino acid substitutions within conserved regions of *WOX*-like proteins. These substitutions may underlie the smooth phenotype observed in *Cylactis* species (Figure S4). This in-depth analysis across multiple *Rubus* genomes and allelic variations provides significant insights into the molecular mechanisms underlying prickleless phenotypes and offers pathways for targeted breeding in commercial *Rubus* fruit crops.

While investigating *WOX* genes in *Rubus*, we noted a few unexpected gene absences (Fig. 3f). In the *R. argutus cv.* Hillquist genome we were unable to find the *WUS* ortholog, but it was found in the *R. ulmifolius* cv. Burbank assembly. The surrounding genes are present on chromosome 1 in the cv. Hillquist assembly indicating the absence is from a loss of the gene in *R. argutus* or an issue with the assembly. Similarly, we were unable to find the *WOX2* gene for *R. idaeus* in the cv. Anitra assembly but did find it in the cv. Joan J. assembly. These observations underscore a broader challenge in characterizing gene families across plant genomes, where distinguishing true gene loss from assembly gaps remains an important consideration when inferring gene family content and evolution.

### Transformation and confirmation of *S* gene

We identified specific alleles of the *RuWOX1*/*RiWOX1* gene that contribute to the prickleless trait observed in several modern blackberry and raspberry cultivars. Although prickleless raspberry and blackberry cultivars are available, many elite cultivars, including PW-D0760, remain prickled. To verify that *RuWOX1* is causative of the prickleless phenotype and demonstrate the potential to enhance commercial lines, we developed a gene-editing approach to knock out *RrWOX1* (*Rubus* subg*. Rubus WOX1* in Blackberry) in PW-D0760. Since PW-D0760 is triplex for the *Ruwox1-1* allele (Fig. 3e), editing just the single wild-type copy of *RrWOX1* could yield the desired prickleless trait.

**Figure 4:**
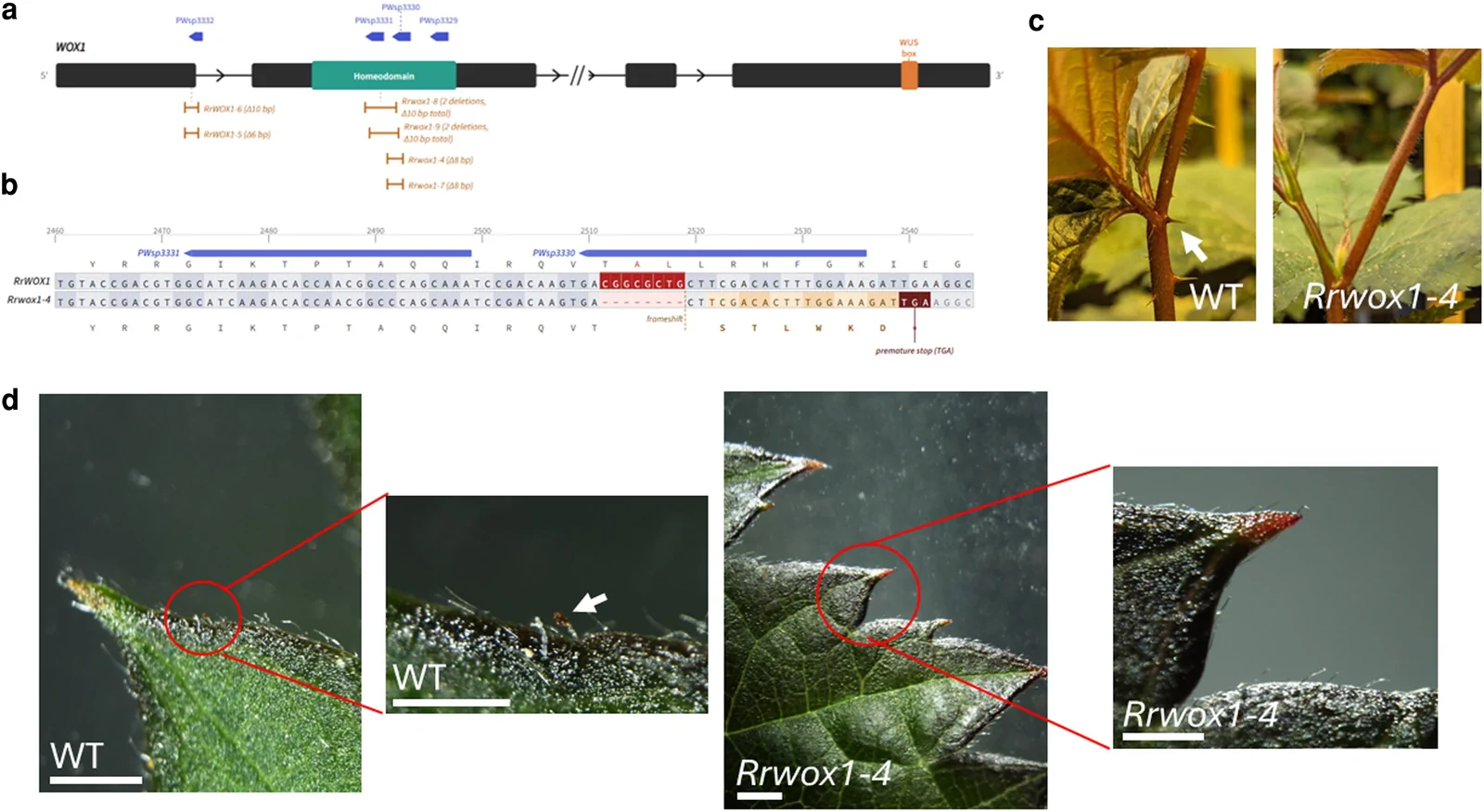
Edited alleles and mutant phenotype. a) *Rubus* wild-type *WOX1* gene structure. The spacer design is labeled green; the location of the mutant alleles is labeled red. The location encoding for 2 domains, the homeodomain and WUS box, characteristic of the WOX family is labeled in blue. b) *Rrwox1-4* allele is aligned to wild-type sequence showing an 8-bp deletion leading to a premature stop codon. c) Absence of prickles with *Rrwox1-4* allele in PW-D0760. Arrow indicates prickle in wild-type PW-D0760. d) Absence of glandular trichomes with *Rrwox1-4* allele in PW-D0760. Arrow indicates prickle in wild-type PW-D0760. Scale bars in first and third images represent 1 mm; bars in second and fourth represent 0.5 mm.

We implemented 2 gene-editing strategies targeting the *RrWOX1* gene in PW-D0760. In the first approach, we designed 3 guide RNA spacers (PWsp3329, PWsp3330, PWsp3331) to target a conserved region of *RrWOX1*, ensuring all haplotypes in PW-D0760 would be edited (Fig. 4a). For the second approach, we designed a specific spacer (PWsp3332) targeting only the wild-type copy of *RrWOX1* in PW-D0760, allowing precise knockout of only the functional allele (Fig. 4a).

Since blackberry had not previously been transformed, we also developed a transformation system specifically for this purpose. Through these strategies, we successfully created several edited alleles in PW-D0760. The first *Rubus* subg*. Rubus–*edited allele, named *Rrwox1-4*, involved an 8-bp deletion within the homeodomain, resulting in a premature stop codon within this critical region (Fig. 4b). The *Rrwox1-4* plants exhibited both prickleless and glandular trichome-free phenotypes, confirming the targeted gene's influence on these traits (Fig. 4c, d).

In addition to *Rrwox1-4*, we generated 5 additional alleles in PW-D0760, each carrying mutations that introduced premature stop codons within or before the homeodomain motif (Table 7; Fig. 4a). All edited plants exhibited the prickleless, glandular trichome-free phenotype, without any other notable phenotypic differences compared to the prickled, wild-type PW-D0760. Both edited and wild-type PW-D0760 plants maintained simple trichomes, suggesting that *RrWOX1* selectively influences the development of prickles and glandular trichomes without impacting other trichome types.

**Table 7:** Alleles in Blackberry *WOX1.*

Name	Source	Polymorphism	Location within gene
Ruwox1-1^a^	*R. ulmifolius* cv. Merton Thornless	8-bp insertion	Homeodomain encoding region
Ruwox1-2^b^	*R. ulmifolius* cv. Burbank Thornless	4-bp insertion	WUS box encoding region
Riwox1-3^c^	*R. idaeus* cv. Burnetholm	8-bp insertion	2nd exon
Rrwox1-4^d^	*Rubus* subg. *Rubus*	8-bp deletion	Homeodomain encoding region
Rrwox1-5^d^	*Rubus* subg. *Rubus*	6-bp deletion	1st exon/intron boundary
Rrwox1-6^d^	*Rubus* subg. *Rubus*	10-bp deletion	1st exon
Rrwox1-7^d^	*Rubus* subg. *Rubus*	8-bp deletion	Homeodomain encoding region
Rrwox1-8^d^	*Rubus* subg. *Rubus*	9-bp/1-bp deletions	Homeodomain encoding region
Rrwox1-9^d^	*Rubus* subg. *Rubus*	5-bp/5-bp deletions	Homeodomain encoding region

^a^ *s* Allele in modern prickleless blackberries derived from *R. ulmifolius*.

^b^ *s* Allele retained in the unphased Burbank Thornless (*R. ulmifolius*) genome assembly.

^c^ *s* Allele in modern prickleless red raspberries (*R. idaeus*).

^d^Novel *s* alleles created through gene editing resulting in the prickleless phenotype in the tetraploid blackberry (*Rubus subg*. *Rubus*) PW-D0760.

### Alternate targets in blackberry

Having identified *WOX1* as the causal gene at the *s* locus, we next asked whether the broader regulatory network controlling prickle initiation in *Rubus* is shared with the canonical Arabidopsis trichome network. Prior work in *Rosa hybrida* showed that orthologs of core Arabidopsis trichome regulators—including *GL1*, *GL3*, *TTG1*, and *TTG2*—are expressed at higher levels in prickles than in bark, and in prickled relative to prickle-free bark (Swarnkar et al. 2021), raising the possibility that loss of these regulators is sufficient to abolish prickle formation in Rosaceae.

A primary candidate from earlier GWAS and expression analyses of a segregating *s^ri^* population in red raspberry was *MYB16-like* (Ra_g19351), a MIXTA-like R2R3-MYB that regulates conical cell outgrowth and trichome initiation (Khadgi and Weber 2020b; Khadgi and Weber 2020c). However, sequencing of prickleless blackberry and red raspberry genomes revealed no loss-of-function mutations in *MYB16-like*, and CRISPR knockouts produced no visible phenotype (Figure S5a), excluding it from further analysis.

We next targeted the core trichome regulator *AtGL1*, identifying the single blackberry ortholog (Ra_g21218) and recovering edited PW-D0760 events carrying 4 unique loss-of-function alleles; (Figure S5b). Contrary to expectations under a conserved-network model, *Rugl1* mutants retained prickles, glandular trichomes, and simple trichomes on leaves and canes. These negative results indicate that, despite the elevated expression in prickle tissue in rose, loss of the trichome canonical regulator is individually insufficient to disrupt prickle or trichome development in *Rubus*, arguing against a simple recapitulation of the Arabidopsis network.

To test whether GL1-family activity is nonetheless involved but masked by paralog redundancy, we transformed PW-D0760 with a *p35S::GL1-SRDX* construct, in which *RuGL1* is fused to the SRDX dominant repression domain to suppress targets shared with redundant paralogs (Hiratsu et al. 2003). T0 events lacked prickles and glandular trichomes but retained simple trichomes (Figure S5c). Together, these results indicate that GL1-family transcription factors do contribute to prickle and glandular trichome differentiation in *Rubus*, but that the relevant activity is distributed across redundant paralogs rather than residing in a single canonical ortholog—a divergence in network topology from Arabidopsis that has direct implications for breeding strategies targeting prickleless cultivars.

## Discussion

We have identified the genetic basis of the prickleless trait in both red raspberry and blackberry. Previous mapping efforts using single reference genomes resolved the locus to chromosome 4 (Khadgi and Weber 2020b; Johns et al. 2025).

By integrating multiple *Rubus* genomes—including a new assembly of *R. ulmifolius* cv. Burbank Thornless, along with *R. argutus* cv. Hillquist (Brůna et al. 2023), *R. idaeus* cv. Anitra (Davik et al. 2022), and *R. occidentalis* “ORUS 4115-3' (VanBuren et al. 2016)—with GWAS, BSA, and IBD analyses, we refined the locus to <400 kb. Because the *S* locus is allelic in blackberry and red raspberry (Lewis 1939; Jennings 1986, 1988), we combined cultivars from both species to identify precise recombination boundaries. Using the combination of genomic analyses on both sources of the *S* allele reduced the size of the physical region to less than 400 kb.

To prioritize candidates within the 400-kb region, we used RNA sequencing (RNA-seq) on a self-crossed PW-D0007 population, segregating for the prickleless trait. Of the 62 genes within our identified region, 5 genes had altered expression. One of these, Ra_g19519, showed a 2- to 3-fold reduction in expression and was annotated as belonging to the *WUSCHEL-Related Homeobox* (*WOX*) family. *WOX* genes encode a phylogenetically conserved group of homeodomain transcription factors (van der Graaff et al. 2009). Named after the archetypal *WUSCHEL* (*WUS*) gene, which is essential for stem cell niche maintenance within the shoot apical meristem, *WOX* genes regulate meristematic activity, organogenesis, and spatial patterning across various plant tissues (Laux et al. 1996). We found that Ra_g19519 was most closely related to the Arabidopsis gene *AtWOX1*. However, using sequence similarity and synteny we showed that another gene, Ra_g20143, was the most direct ortholog of *AtWOX1* in blackberry. Within the Rosaceae, the subclade of Ra_g19519 is present in *Fragaria*, *Rosa*, and *Rubus,* but not in *Prunus.* This suggests an expansion of the *WOX1* subclade within the Rosoideae subfamily of the Rosaceae. In *Rosa hybrida*, gene expression has been studied in epidermal cells at different stages of prickle development (Swarnkar et al. 2021). Notably, *RhWOX1*, which is the ortholog of Ra_g19519, (RcHm_v2.0_Chr7g0178511) is ∼20-fold upregulated in growing rose prickle epidermis relative to initiating epidermis or hardened prickles (Figure S6). *AtWOX1* acts as a transcriptional repressor promoting cell proliferation in the leaf lamina, partly through auxin biosynthesis, and is genetically redundant with *AtWOX3* (Ikeda et al. 2009; Zhang et al. 2011, 2020b; Nakata et al. 2018). *WOX* genes in this broader clade have been independently implicated in epidermal outgrowth and lateral patterning across diverse species: *SmWOX3* in eggplant prickle loss (Nakata et al. 2012; Qian et al. 2021), *OsWOX3* in rice trichome formation (Sun et al. 2017), maize *NS1/NS2* in leaf margin founder cells (Nardmann et al. 2004), *CsWOX3* in cucumber fruit spines (Xu et al. 2024), citrus *CsWUS* in thorn-versus-branch fate (Zhang et al. 2020a), and *WS1* in lettuce spine development (Sun et al. 2024).

Manual annotation within the *S* locus led to the identification of mutations within Ra_g19519 orthologs RuBurbank_v1_000000F_038, in blackberry, and Ri04_g21623, in red raspberry. Our findings reveal that an 8-bp insertion in the second exon of *RuWOX1*, termed *Ruwox1-1*, introduces a premature stop codon in the homeodomain, likely leading to a nonfunctional protein and the subsequent prickleless phenotype. We also found an 8-bp insertion in red raspberry, termed *Riwox1-3*, just after the homeodomain in Ri04_g21623 that also likely leads to a nonfunctional protein. The *Ruwox1-1* and *Riwox1-3* alleles co-segregate with the prickleless (*s^ru^*, *s^ri^*) alleles across the respective populations analyzed, strongly suggesting that these mutations are responsible for the loss of prickles.

The *R. ulmifolius* cv. Burbank Thornless genome provided additional insights into the genetic complexity of the prickleless trait. In an earlier GWAS of 374 blackberry genotypes grown by the University of Arkansas Division of Agriculture, Burbank Thornless was the only prickleless genotype that was heterozygous for the Single-nucleotide polymorphism (SNP) most strongly associated with prickleless canes at 33,636,565 bp on chromosome Ra04 (Johns et al. 2025). We found that Burbank Thornless carries 2 distinct *RuWOX1* alleles: *Ruwox1-1* and *Ruwox1-2*, the latter characterized by a 4-bp insertion in the WUS box and other SNPs. Compound heterozygosity for 2 loss-of-function alleles likely explains the prickleless phenotype despite heterozygosity at the locus. These findings highlight the importance of understanding the interaction between different alleles at the *RuWOX1* locus, as they have significant implications for *Rubus* breeding programs aiming to develop prickleless cultivars. Because the Burbank Thornless assembly is unphased, only one haplotype at the *S* locus was represented in the final reference sequence. In this case, the assembled haplotype corresponds to *Ruwox1-2*, while the alternative allele (*Ruwox1-1*) was carried forward into modern prickleless breeding germplasm. This underscores how reference assemblies may not always capture the functionally relevant allele and highlights the importance of haplotype-resolved assemblies for connecting reference genomes to functional alleles.

In the commercial blackberry line PW-D0760, the presence of the *Ruwox1-1* allele in triplex provided further validation of the gene's role in prickle formation. Our gene-editing experiments in PW-D0760, designed to knock out *RrWOX1*, were successful. We generated several alleles carrying premature stop codons, with all resulting plants exhibiting the prickleless and glandular trichome-free phenotypes. These edited lines lacked glandular trichomes, suggesting that *RuWOX1* may regulate both prickle and glandular trichome development. No other phenotypic differences were observed between wild-type and edited plants, indicating that *RuWOX1* has a specific role in prickle and glandular trichome formation without affecting other trichome types or overall plant morphology.

Within our diversity panel, varieties that exhibited prickles always had glandular trichomes suggesting that these epidermal outgrowths share common developmental pathways. Our work indicates that *RuWOX1* could be a key regulatory gene influencing both traits. However, varieties that had glandular trichomes had a moderate correlation with the presence of prickles (r = 0.47). This suggests other factors are involved with prickle formation and further research is necessary to fully understand the genetic networks governing the formation of prickles and glandular trichome development.

Our findings provide a clear case for parallel evolution at the molecular level, where the prickleless trait arose from at least 3 different natural mutations (*Ruwox1-1*, *Ruwox1-2*, *Riwox1-3*) all in the same *Rubus WOX1* gene, even in distantly related *Rubus* species. This repeated independent targeting suggests *WOX1* acts as a key developmental hotspot for this trait. Such parallel evolution is often favored when a single gene acts as a developmental choke point with low pleiotropy, where it represents an efficient evolutionary pathway to alter a specific trait without incurring major fitness penalties. Our own gene-editing results support this, as the *Rrwox1-4* knockout plants were phenotypically normal, aside from the targeted loss of prickles and glandular trichomes. The reduced risk of pleiotropy makes *WOX1* an excellent target for crop improvement. At the same time, a recent study in *Solanum* revealed that prickle loss has repeatedly evolved through mutations in a *LONELY GUY* (*LOG*) cytokinin biosynthesis gene, known as *prickleless* (*PL)* (Satterlee et al. 2024). At least 16 independent *log* mutations were identified across wild and domesticated species, and similar mechanisms were found in unrelated plants like barley, rice, jujube, and rose. The *PL* gene was shown to be a conserved ortholog shared across angiosperms, including *Rubus*.

In Arabidopsis a member of the *LOG* gene family, *AtLOG4*, has been shown to promote expression of *AtWUS* in the shoot apical meristem (Chickarmane et al. 2012). A similar genetic interaction between *RuLOG* and *RuWOX1* may be conserved in glandular trichomes and prickles meristem formation. Gene editing of *PL* successfully removed prickles from eggplant without affecting other traits, highlighting a convergent, genetically simple mechanism that could be applied in *Rubus* breeding (Satterlee et al. 2024; Zhang et al. 2024). While *WOX1* is a confirmed pathway in *Rubus*, other candidate gene targets such as *LOG* could be examined to achieve loss of prickles in *Rubus*, possibly without loss of glandular trichomes.

Genome editing of RuWOX1 offers a direct route to prickle-free commercial cultivars. This is particularly valuable in blackberry, where the recessive *s^ru^* locus is in linkage with several unfavorable traits—susceptibility to winter injury, trailing habit, late harvest, large seeds, and high fruit acidity (Moore 1984)—and where intensive selection has produced an extensive linkage disequilibrium block around *s^ru^* that makes linkage drag difficult to break by crossing. Editing allows breeders to introduce loss-of-function alleles directly into elite prickled backgrounds without inbreeding or extensive backcrossing.

Future work should focus on further characterizing the regulatory role of *RuWOX1* in epidermal development and determining how this gene interacts with other pathways involved in trichome and prickle formation. Additionally, the development of more robust transformation systems for *Rubus* species will facilitate the broader application of gene-editing technologies, accelerating the development of improved cultivars with desirable traits such as pricklelessness, disease resistance, and enhanced fruit quality.

## Material and methods

### Vector design

The binary plasmid, pWISE8807, was designed to express the *Lachnospiraceae bacterium* ND2006 Cas12a (LbCas12a) endonuclease (Zetsche et al. 2015) and 3 guide RNA spacers (PWsp3330: CCTCGCGCGGTGGTTCTGAAACC, PWsp3331: CTGGGCCGTTGGTGTCTTGATGC, PWsp3332: CGTATGTACGTACCCTGATGCTG) to knock out the functional copy of the *WOX* gene in *PW-D0760*. PWsp3330 and PWsp3331 target exon 2 of the gene, which encodes the homeodomain, for two reasons: (i) in-frame mutations or frameshifts are likely to produce loss-of-function alleles; (ii) the loss-of-function mutations in *Ruwox1-1* and *Riwox1-3* also fall within this same region. PWsp3332 was designed to target the first exon to target only the wild-type haplotype of *RrWOX1*; it contains one mismatch to the sequence of the *Ruwox1-1* allele. All spacers were designed to specifically target the WOX1 gene (Ra_g19519) in PW-D0760, with a minimum of 6 mismatches to the nearest off-target genomic sequence (Table S8). Guide RNA spacers were designed in Benchling (https://www.benchling.com) using the implementation of an off-target specificity score (Hsu et al. 2013). Guides were expressed from the 3′ UTR of the *Ds.RED-Express2* gene (Strack et al. 2008) driven by the *OsAct1* promoter. Transcription of guide RNAs was terminated with the *StPinII* terminator. The guide cassettes were synthesized by GenScript and inserted in the binary plasmid with the *LbCas12a* expression cassette. A disarmed *Agrobacterium tumefaciens* strain was used to introduce a transfer DNA (T-DNA) from the binary plasmid. The T-DNA cassette expresses a plastid-targeted *NptII* expression cassette as a plant selectable marker, the cassette for LbCas12a endonuclease, *DsRED-Express2* with 3′ UTR guide RNA cassette, and a *ZsGreen* expression cassette for transgenic color marker selection. The binary plasmid was re-extracted from *Agrobacterium* and verified by whole plasmid sequencing by plexWell PRO (seqWell, Beverly, Massachusetts, United States).

To generate a dominant-negative GL1 allele, the GL1 coding sequence (lacking its stop codon) was fused in-frame at its 3′ end to a 36-bp sequence encoding the 12-amino-acid SRDX repression domain (LDLDLELRLGFA), an engineered EAR motif derived from the C-terminal region of Arabidopsis SUPERMAN that converts transcriptional activators into dominant repressors of their target genes, including in the presence of functionally redundant endogenous activators (Hiratsu et al. 2003). The resulting *GL1-SRDX* fusion was placed under the control of the CaMV 35S promoter with a synthetic 5′ UTR (synJ) and terminated by the *PinII* terminator, yielding the p35S:GL1-SRDX expression cassette used for plant transformation.

### Plant transformation

Blackberry nodal explants were harvested from the greenhouse and sterilized using ethanol and bleach, followed by sterile water rinses. Sterile nodes were then cultured on a propagation medium for 3 to 6 wks prior to transformation. Explants excised from in vitro shoot cultures were inoculated with *Agrobacterium* prior to co-culture on sterile filter paper for 5 to 6 d and were then transferred to a selective medium. Two transformation methodologies were developed in which initial regenerants were usually chimeric, containing a mixture of nontransformed and transformed edited cells. To obtain uniformly edited plants, an additional round of regeneration was induced in vitro from various chimeric tissues resulting in the recovery of edited plants suitable for characterization. We refer to this process as “chimera cleanup.” Using this approach, multiple uniform and uniquely-edited plants could be recovered from each chimera. Up to 6 uniquely-edited plants have been recovered from the cleanup of a single chimera. Time in culture posttransformation ranged between 3.5 to 8 months before plants suitable for characterization were rooted and acclimated to greenhouse conditions. The detailed information about transformation is in PCT/US2025/032956 and PCT/US2025/033708.

### Trichome and prickle phenotyping

Accessions used in the study were accessed through the USDA-ARS National Clonal Germplasm Repository (NCGR) at Corvallis, Oregon (Table S1). Leaves with petioles attached were collected from all included accessions and observed under a dissecting microscope. Accessions were grouped into 4 classes based on presence and abundance of various trichome types: (i) simple trichomes only, (ii) simple trichomes and short (<1 mm in length) glandular trichomes, (iii) simple trichomes, short glandular trichomes, and sparse elongated (>1 mm in length) glandular trichomes, or (iv) simple trichomes, short glandular trichomes, and abundant (>20/cm of tissue) elongated glandular trichomes.

Presence/absence of prickles on petioles was also assessed. We observed variation in epidermal structures, where many lines had thin, nonlignified epidermal outgrowths we termed “bristles.” Under microscopy, we observed that these bristles were elongated glandular trichomes, which could be differentiated from prickles by their thickness and presence of a glandular head. We scored plants according to large epidermal outgrowth types, assigning plants into one of 4 groups: (i) lack of “bristles” or prickles, (ii) “bristles” only, (iii) prickles only, or (iv) both “bristles” and prickles. Prickle observations on petioles were cross-checked against observations from canes, and observation of a prickle in either organ resulted in the line being grouped as prickled.

### *R. ulmifolius* cv. Burbank Thornless genome sequencing, assembly, and annotation

The diploid *R. ulmifolius* cultivar “Burbank Thornless” (PI 554060) was obtained from the USDA-ARS NCGR for genome sequencing. Nuclear flow cytometry with *Vinca major* as a standard estimated the genome size at ∼341 Mb. High-molecular-weight DNA extracted from young leaves was sequenced with PacBio Sequel (6 SMRT cells). Illumina Hi-C libraries were prepared from young leaf tissue, while RNA from root tips, leaves, and stems was used for Illumina RNA-seq and PacBio Iso-Seq. PacBio long reads were assembled with FALCON/FALCON-Unzip, and undercollapsed heterozygosity was resolved with Purge Haplotigs (Chin et al. 2016; Roach et al. 2018). Scaffolding was performed with Hi-C data using Juicer/3D-DNA followed by manual polishing with Juicebox Assembly Tools (Durand et al. 2016; Dudchenko et al. 2017, 2018). The final assembly comprised 7 chromosome-scale scaffolds, which were ordered and oriented against the *R. argutus* cv. Hillquist reference genome.

For structural annotation, transcript assemblies were generated from RNA-seq and Iso-Seq data and combined with protein homology from 24 diverse plant species and curated databases. Gene models were predicted using multiple ab initio and evidence-based approaches (AUGUSTUS, FGENESH, EXONERATE, PASA) and refined by PASA to add UTRs, splice site corrections, and alternative isoforms (Salamov and Solovyev 2000; Slater and Birney 2005; Stanke et al. 2006). Low-confidence models overlapping repeats or lacking transcript/homology support were filtered. Functional annotation was assigned using BLAST searches against SwissProt, Araport11, and NCBI databases, InterProScan domain analysis, and KEGG/eggNOG orthology (Zdobnov and Apweiler 2001; Moriya et al. 2007; Camacho et al. 2009; Huerta-Cepas et al. 2017; Afgan et al. 2018). Complete methods describing genome assembly and annotation are provided in Supplemental File S1.

### Genome-wide association study

The software package GWASpoly was used to perform genome-wide association mapping (Rosyara et al. 2016). Genomic DNA was extracted with Qiagen DNeasy Plant Mini Kit, and sequencing was performed with the NovaSeq 6000 instrument with 150-bp paired end reads to approximately 30× coverage. Initially, reads were cleaned with HTStream (Petersen et al. 2015), mapped to the *R. argutus* cv. Hillquist reference genome with the aligner “bwa mem” (Li and Durbin 2010), and variants were called with freebayes in tetraploid mode “-p 4” (Garrison and Marth 2012). The variant set was randomly down-sampled to 1 million SNPs then converted to allele dosage format with the GWASpoly function “VCF2dosage” with the following filters: min.DP = 2, max.missing = 0.05, min.minor=5. The random down-sampling was applied in lieu of linkage disequilibrium (LD)-based SNP pruning owing to the challenge of estimating LD in tetraploids. After filtering, 333,198 SNPs remained for analysis. Marker significance scores were computed with the “GWASpoly” function using the “1-dom” tetraploid model with binary prickle/no-prickle trait scores, and *P* values were adjusted with a Bonferroni correction. A maximum genotype frequency threshold of 1 to 5/N was applied during model fitting, as recommended by the GWASpoly authors. The analysis included 29 prickleless and 14 prickle control samples. Given that the reference genome (Hillquist) contains the dominant allele for the prickle trait, the 1-dom-ref marker scores were used for the genome scan Manhattan plot, which included a Bonferroni correction of *P* values with an alpha cutoff of 0.05. To assess potential effects of population structure, we additionally ran the association analysis with K-model approach via a LOCO method implemented in GWASpoly. We then compared the QQ-plots with and without the LOCO method.

### BSA

We generated a segregating population by self-crossing PW-D0007, a line which was determined to be triplex for the “*s*” allele (*Ssss*). The resulting progeny displayed either the prickleless (32 individuals) or prickled phenotype (65 individuals). We used this population in a BSA. For each bulk, DNA was extracted with DNeasy Plant Mini Kit and pooled. High-throughput sequencing was then performed on both groups with the NovaSeq 6000 instrument with 150-bp paired end reads. We generated ∼330× genome-wide sequence data representing the genetic makeup of the prickleless and prickled groups. The reads were cleaned with HTStream (Petersen et al. 2015) mapped to the *R. argutus* cv. Hillquist genome assembly with the aligner “bwa mem” (Li and Durbin 2010), and variants were called with freebayes (Garrison and Marth 2012). To identify genomic regions associated with the prickleless trait, the variants were filtered by allele frequency to match expectations of the recessive inheritance: prickleless bulk allele frequency ≥ 0.95 and control bulk allele frequency ≤ 0.85. The number of filtered variants per 100-kbp window with a 50-kb step size were then plotted to identify regions associated with pricklelessness.

### IBD

Whole genome sequencing reads obtained for GWAS were aligned to the *R. argutus* cv. Hillquist, *Rubus occidentalis* v3, and *R. idaeus* cv. Anitra genomes and visualized in the jbrowse genome viewer. Syntenic regions of the 3 genomes were identified initially by selecting several genes within the *R. argutus* cv. Hillquist genome and running BLAST with the other genomes as the subject. Regions containing the resulting BLAST hits were then aligned with the regions from the other genomes. Most gene models from the region of one genome were seen in the others with high sequence conservation. SNPs correlating with differences in the prickle phenotype were checked in the other genomes. Based on SNP density in the region and the sequence diversity among the cultivars, a region of 20 kbp was scanned in each direction any time a break point in one cultivar was assumed to reduce the chance of calling a false break point. SNPs associated directly with the prickleless phenotype were required to be homozygous in the prickleless cultivars and no more than heterozygous in the prickled cultivars. Because the *R. idaeus* cv. Anitra is prickleless, SNP calling was adjusted when comparing in that context. The reverse complement of the region from the *R. occidentalis* was used when aligning the regions.

### Orthology search for candidate gene

The genomic sequence of Ra_g19519 was used to find orthologs in *Prunus avium* (Shirasawa et al. 2017), *Rubus occidentalis* v3 (VanBuren et al. 2016), *R. argutus* cv. Hillquist (Brůna et al. 2023), *R. ulmifolius* cv. Burbank thornless (this work), *Rubus* L. subg. *Rubus* Watson (Paudel et al. 2025), *R. idaeus* cv. Anitra (Davik et al. 2022), *R. idaeus* cv. Joan J., (Zhou et al. 2023), and *Arabidopsis thaliana* Araport11 (Cheng et al. 2017). Additional searches using Arabidopsis *WOX* genes as query were conducted to find additional family members. Results from Hmmer3 (Eddy 2008, 2009, 2011) and BLAST (Camacho et al. 2009) searches were aligned using MAFFT (Katoh et al. 2002; Katoh and Standley 2016) and Clustalo (Sievers et al. 2011; Sievers and Higgins 2018), for DNA and protein sequences, respectively. The DNA from conserved exons were selected including insertions and deletions to keep mutants close to their nonmutant relatives. UPGMA consensus trees were built with the alignments using a Jukes–Cantor distance model and 500 bootstrap resampling (Jukes and Cantor 1969).

### RNA sequencing

For RNA analysis between our BSA prickles/prickleless samples, the tips of blackberry plants were excised in the greenhouse, immediately submerged in RNA Later (Invitrogen), placed on ice, and transported to a 4 °C refrigerator, where they were stored for 24 h to ensure thorough penetration of the RNA Later into the tissue. Meristems were then carefully dissected under a stereomicroscope and stored at −20 °C until RNA extraction. RNA was extracted using the QIAGEN RNeasy Plant Mini Kit, incorporating 2 apical meristems per sample. The extraction buffer was supplemented with 2.5% polyvinylpyrrolidone-40 (PVP-40) (PhytoTech Labs), and samples were incubated at 65 °C for 10 min following grinding and buffer addition. An additional wash-step was included in the protocol, and RNA was eluted in 50 µL of nuclease-free water (Invitrogen).

HTStream (Petersen et al. 2015) was used to check reads for quality and prepare for mapping. Hisat2 (Kim et al. 2019) was used to map reads to the *R. argutus* cv. Hillquist genome. The lowest mapping rate for the samples was 84.5%. Htseq-count (Anders et al. 2015) was used to count transcript abundance as counts for prickleless and prickled samples were analyzed with DEseq2 (Love et al. 2014).

## Supplementary Material

kiag551_Supplementary_Data

## Data Availability

Raw PacBio and IsoSeq sequencing data and the genome assembly of *R. ulmifolius* presented here are available at the NCBI under Bioproject ID PRJNA1263752. Hi-C data are available on Bioproject PRJNA512907 (Biosample SAMN14122117; SRA SRX7735966). Illumina transcriptome data are available on Bioproject PRJEB36280 (BioSamples ERX5171502, ERX5171501, ERX5171495, ERX5171494). Interactive Hi-C contact maps of the Burbank Thornless genome sequence assembly are available via https://t.3dg.io/burbank-blackberry-Fig-1 and via the www.dnazoo.org website (https://www.dnazoo.org/assemblies/rubus_ulmifolius). The Burbank Thornless genome assembly and annotation can also be accessed at the Genome Database for Rosaceae (Publication datasets | GDR) under the accession number tfGDR1091 and on phytozome (https://phytozome-next.jgi.doe.gov/info/Rulmifolius_v1_1).
